# Young Infants' Neural Processing of Objects Is Affected by Eye Gaze Direction and Emotional Expression

**DOI:** 10.1371/journal.pone.0002389

**Published:** 2008-06-11

**Authors:** Stefanie Hoehl, Lisa Wiese, Tricia Striano

**Affiliations:** 1 Neurocognition and Development Group, Max Planck Institute for Human Cognitive and Brain Sciences, Leipzig, Germany; 2 Hunter College, City University of New York (CUNY), New York, New York, United States of America; New York University, United States of America

## Abstract

Eye gaze is an important social cue which is used to determine another person's focus of attention and intention to communicate. In combination with a fearful facial expression eye gaze can also signal threat in the environment. The ability to detect and understand others' social signals is essential in order to avoid danger and enable social evaluation. It has been a matter of debate when infants are able to use gaze cues and emotional facial expressions in reference to external objects. Here we demonstrate that by 3 months of age the infant brain differentially responds to objects as a function of how other people are reacting to them. Using event-related electrical brain potentials (ERPs), we show that an indicator of infants' attention is enhanced by an adult's expression of fear toward an unfamiliar object. The infant brain showed an increased Negative central (Nc) component toward objects that had been previously cued by an adult's eye gaze and frightened facial expression. Our results further suggest that infants' sensitivity cannot be due to a general arousal elicited by a frightened face with eye gaze directed at an object. The neural attention system of 3 month old infants is sensitive to an adult's eye gaze direction in combination with a fearful expression. This early capacity may lay the foundation for the development of more sophisticated social skills such as social referencing, language, and theory of mind.

## Introduction

Social referencing is the ability to search for and to use social signals in order to modulate behavior in new or ambiguous situations [1]. Adults constantly make use of social signals like emotional expressions to guide behavior in ambiguous or dangerous situations [e.g. 2], [3]. Often this is done without conscious control or cognitive effort. For instance, fearful faces which may signal threat automatically captures attention [4]–[6]. An important neural structure underlying this social threat detection system is the amygdala which is sensitive to fearful expressions and also to eye gaze direction in angry and fearful faces [7], [8]. However, the developmental trajectory that leads to the efficient detection of relevant social signals in human adults has only been investigated in parts.

For decades research showed that infants show social referencing behavior by the end of the first year [see 1 for a review]. For instance, when faced with an ambiguous and potentially dangerous situation, infants turn to their caregivers and use referential emotional cues to adjust their behaviour [9]–[11]. Importantly, the majority of studies in this field have explored infants' behavioral responses toward an ambiguous or threatening stimulus as a function of an adult's emotional expression and often infants were required to locomote [9]–[11]. These measures are highly difficult to apply with younger infants, whose scope of actions is limited. However, it is conceivable that infants' attention system can be affected by referential emotional signals even before infants are able to respond on a behavioral level.

Previous research has demonstrated young infants' remarkable social skills. For example, newborns differentiate between direct and averted eye gaze [12]. By 3 months, infants are able to follow another person's eye gaze [13]. At around the same age, shifts of eye gaze bias infants' attention toward cued targets and facilitate encoding of cued objects [14]–[16]. Infants are also sensitive to emotional expressions in face and voice from a very early age onwards [17]–[19]. For instance, the positive slow wave of the infant ERP is sensitive to information conveyed by emotional expressions and eye gaze in 4-month-olds [20]. Another important component in the context of infant face processing is the mid-latency negative central (Nc) component on fronto-central channels. The Nc has consistently been related to attentional orienting to salient stimuli [21], [22], and its amplitude is closely associated with attention as measured by heart rate deceleration [23]. In prior ERP studies, the Nc was sensitive to emotional expressions. An enhanced Nc was found for fearful relative to happy faces [24], angry relative to happy faces [25] and angry relative to happy or neutral prosody [26]. Further, an enhanced Nc was found for angry faces with direct compared to averted eye gaze [27]. Together these results indicate that the Nc response is enhanced by threat-related emotional stimuli.

However, no studies have investigated yet whether young infants' neural system is sensitive to another person's expression of fear toward an object. This involves not only perceiving and discriminating the emotional expression. It also requires that infants link the emotional expression to an unfamiliar object on the basis of eye gaze direction. Possibly, this involves an explicit understanding that the emotional expression is aimed at something in the environment in a referential way. However, rather automatic mechanisms are also conceivable. Based on behavioural studies, the understanding that eye gaze is referential has been attributed to infants by 8 to 12 months of age [28], [29]. Referential understanding of emotions has been demonstrated by 12 months of age [10].

**Figure 1:**
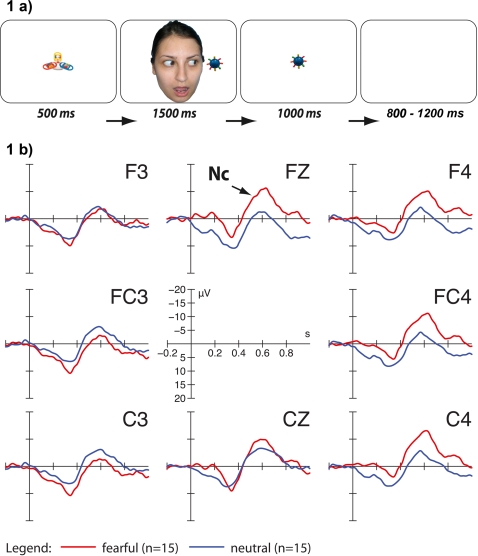
Stimuli and results for study 1. Example of one trial in the fearful face condition (Figure 1a). A central attractor object preceded each trial to catch infants' attention. Then a fearful or neutral face was presented looking at an object. A blank screen period followed after the face and object stimulus (not depicted, varying duration between 400 and 600 ms). The same object was then presented alone, followed by a blank screen period. Note that the depicted face was not used in the current study. Original faces cannot be published. ERP responses to objects alone on fronto-central channels (Figure 1b). Note that negative is plotted upwards. Objects that had before been gaze cued by a fearful expression elicited a substantially enhanced Nc component (red) compared to neutrally cued objects (blue).

**Figure 2:**
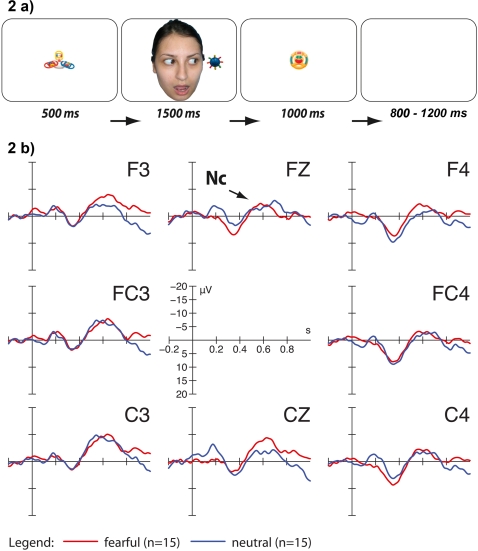
Stimuli and results for study 2. Example of one trial in the fearful face condition (Figure 2a). Following each face-object stimulus a different object was presented. ERP responses to objects alone on fronto-central channels (Figure 2b). Note that negative is plotted upwards. No difference was found between Nc amplitude for objects following a fearful compared to a neutral face plus objects dyad.

**Figure 3:**
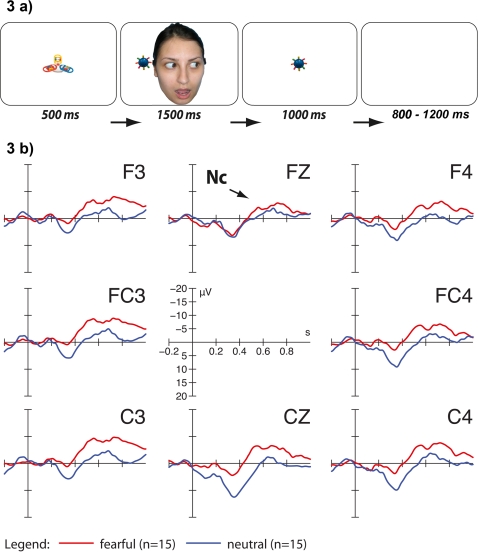
Stimuli and results for study 3. Example of one trial in the fearful face condition (Figure 3a). Neutral and fearful faces gazed away from the objects that were subsequently presented alone. ERP responses to objects alone on fronto-central channels (Figure 3b). Note that negative is plotted upwards. No difference was found between Nc amplitude for objects that had been presented with a fearful compared to a neutral face gazing away from the object.

We directly investigated whether young infants' neural responses can be affected by an adult's fearful expression and eye gaze when directed at an unfamiliar object. To explore this question we chose the measurement of a well-established neural correlate of infants' attention, namely the Nc component of the ERP. Three visual ERP experiments with healthy infants were conducted in order to explore young infants' sensitivity to eye gaze and emotional expressions that are directed at external objects. In study 1, 3-month-old infants were exposed to adult faces looking toward unfamiliar objects while posing either a fearful or a neutral expression. Following each face-object stimulus the respective object was presented again without the face (Figure 1a). We chose to test this very young age group because infants use eye gaze cues to guide attention and facilitate learning by that age [14]–[16]. We hypothesized that infants would react with an enhanced Nc component to objects that had been gaze cued by a fearful compared to a neutral face before. If the infant brain is able to link the emotional expression to the object through eye gaze direction, no effect of emotion on Nc amplitude should be found if (1) following each face-object stimulus a novel object is presented (study 2; Figure 2a) and (2) eye gaze of the adult is averted away from the object (study 3; Figure 3a).

## Results

ERP responses to objects in study 1 are presented in Figure 1b. Objects that had previously been gaze cued by an adult with a fearful expression elicited a substantially increased Nc component on right fronto-central channels when compared to objects that had been looked at by a neutral face.

For statistical analyses mean amplitude was considered within a time window of 500–700 ms after stimulus onset. Mean amplitude was averaged across channels within each region of interest, which were defined as follows: left fronto-central (F3, FC3 and C3), fronto-central (FZ and CZ) and right fronto-central (F4, FC4 and C4). For each of the three studies a repeated measures General Linear Model was applied with emotion (fearful/neutral) and region of interest (left/central/right) as within-subject factors. In study 1, the General Linear Model detected a significant interaction between emotion and region of interest, F_(2,13)_ = 6.63, p = 0.005. Posthoc two-tailed t-tests revealed that on right channel sites the fearful condition elicited the more negative amplitude peak (mean = −9.58; SD = 2.4) than the neutral condition (mean = −2.74; SD = 2.4), t_(14)_ = −2.94, p = 0.011. This difference was not significant on left, t_(14)_ = 0.766, p = 0.456 or central channels, t_(14)_ = −1.41, p = 0.179. Visual inspection of the data suggested that the ERP may be more negative in the fearful condition even before onset of the Nc. Therefore, a peak to trough analysis was conducted with the positive peak of the so-called Pb component (positive before) between 200 and 400 ms and the negative peak of the Nc between 348 and 700 ms. The interaction between emotion and location was still marginally significant, F_(2,13)_ = 3.141, p = 0.060. A t-test on right channel sites also revealed a significant effect, t_(14)_ = −2.735, p = 0.016. The difference between peak of the Pb and trough of the Nc was greater for the fearful (mean = −25.75, SD = 15.0) than for the neutral condition (mean = −21.09, SD = 13.8).

Applying the same General Linear Model and additional t-tests, no effects of emotion or location on amplitude of the Nc were found in studies 2 and 3 with a statistical threshold of p<0.05 (see Fig. 2b and 3b).

We further applied a General Linear Model with emotion (fearful/neutral) and region of interest (left F3, FC3, C3/central FZ, CZ/right F4, FC4, C4) as within-subject factors, and study (1, 2, 3) as a between-subjects factor. This was done in order to test our hypothesis that the factor emotion would have a differential impact on mean amplitude of the Nc in study 1 compared with studies 2 and 3. As expected, a significant interaction between emotion, region of interest and study was found, F_(2,42)_ = 2.506, p = 0.048.

## Discussion

Infants allocated increased attention toward objects that were potentially dangerous, namely objects that had been gaze cued by an adult with a fearful expression. The lateralization of this effect to the right hemisphere is in accordance with previous findings using a similar paradigm with neutral faces [15] and a right-hemispheric bias for face processing in previous ERP studies investigating the Nc component [30].

However, alternative explanations for these results should be taken into account. First, it is conceivable that a fearful face directing eye gaze toward a simultaneously presented object elicits an unspecific arousal which causes infants to direct attention toward any following stimulus. Therefore, in study 2 infants were presented with novel objects after each face-object dyad. No difference was found between conditions in this study which suggests that infants did not generalize the emotional expression to any subsequent stimulus.

Second, it may be that a fearful face attracts infants' attention away from the object more than a neutral expression. Therefore, when presented again, objects that had been gaze cued by a fearful face may be more novel and attract more attention compared to objects that had been accompanied by a neutral face. Further, it may be that infants simply associated the fearful face with the simultaneously presented object without regarding the adult's gaze direction. In study 3, eye gaze of the adult was therefore averted away from the object. Again, no difference between fearful and neutral trials was found. This suggests that infants were indeed sensitive to the adult's eye gaze direction, and did not react with enhanced attention toward objects that had previously been presented with a fearful face gazing away from the object. The results of this study also speak against the interpretation that infants in study 1 allocated more attention toward fearfully cued objects, because these were less familiar. If the presence of a fearful face prevented efficient encoding of objects in study 1 the same effect should have been observed in study 3, which was not the case.

The current experiments are the first to demonstrate 3-month-old infants' sensitivity to fearful expressions together with referential eye gaze. These findings are intriguing considering that previous studies failed to demonstrate social referencing behavior in infants at 10 months [31], [32]. What can account for this discrepancy? Instead of exploring infants' behavioral reactions to displays of emotion directed at novel objects, we chose to measure infants' attention to gaze cued objects as reflected by the Nc component. We show that infants' attention toward novel objects is substantially increased by an adult's expression of fear toward the object. We suggest that even though young infants' scope of action is limited, they are nonetheless already prepared to utilize adults' social signals in order to guide their attentional resources [see also 33], [34].

However, our results leave open whether infants were really aware of the referential meaning of eye gaze and a fearful expression in our experiment. Potentially, the fearful expression elicited an enhanced arousal which was then associated with the gaze cued object. Even without an explicit understanding of the referential meaning of the communicative signals this would lead to enhanced attention toward fearfully cued objects. In either way, we show that remarkably young infants are able to discriminate an emotional from a neutral expression and associate it with a gaze cued object.

The cortical source of the Nc component has been located in the prefrontal cortex and anterior cingulate [35], which is implicated in conflict monitoring and attention control [36]–[40]. In patients with panic disorders abnormal activations have been found in response to fearful facial affect in the anterior cingulate and the amygdala [41]. The amygdala is known to play a crucial role in the processing of threat-related stimuli [42], [43], and is sensitive to the direction of eye gaze in faces displaying fearful or angry affect [7]. A fearful face directing eye gaze at a novel stimulus may rapidly elicit activation in subcortical structures which then modulate activation in cortical structures related to attentional processes [4]. Our findings suggest that this mechanism may come on-line very early in human ontogeny. Indeed, it has been argued that a subcortical face processing pathway, involving the amygdala, exists already in early infancy, and that this pathway modulates responses of cortical areas to social stimuli [44]. This subcortical pathway may be involved in enhancing infants' attention to faces and socially relevant stimuli [45].

Our findings suggest that even before infants are able to regulate behavior according to an adult's emotional signals toward objects or persons, infants' attention system can already be affected by social cues that signal threat, i.e. a fearful face with eye gaze directed at an object. Further research is required to determine whether this effect is restricted to threat-related emotional expressions like fear and maybe anger, or whether any emotional expression may elicit a similar effect compared with a neutral face. Future studies should also use behavioural measures of attention which have already been applied successfully with young infants [14]. If similar findings can be obtained using different measures of attention this might help to pinpoint the underlying mechanisms.

It has been argued that the ability to detect eye gaze may have evolved in order to detect threat from potential predators [46]. Eye gaze detection is a very basic mechanism that can be observed very early in human ontogeny and that has evolved early in phylogeny. Even non-mammalian prey species, such as black iguanas and chickens, are sensitive to the direction of eye gaze [see 47 for a review]. Primates systematically follow the gaze direction of conspecifics [48] and follow a human experimenter's gaze even behind a barrier [49], [50]. From an evolutionary perspective the neural mechanisms examined in this study are highly adaptive as they may directly contribute to survival in potentially dangerous situations.

## Materials and Methods

### Subjects and experiments

Fifteen typically developing infants (age range from 3;0 months to 4;0 months; study 1: average age 112.5 days, 11 males; study 2: average age 103.5 days, 10 males; study 3: average age 112.5 days, 8 males) were included in the final samples of each of the 3 experiments, respectively, after their parents had given written consent. In all, another 58 infants had to be excluded from the three experiments due to fussiness or failing to reach at least 10 artifact-free trials per condition for averaging. This corresponds to the common drop-out rate in infant ERP studies [51]. The mean number of trials that were included for every infant per condition was 19.06.

Each trial consisted of a central attractor object (displayed for 500 ms), a fearful or neutral face plus object stimulus (displayed for 1500 ms), a blank screen period with a randomly varying duration (400–600 ms) and an object (displayed for 1000 ms). Every trial was followed by a blank screen period, whose duration varied randomly between 800–1200 ms. Original pictures of neutral and fearful stimulus faces from one male and one female actor were taken from the NimStim Face Stimulus Set (www.macbrain.org). The irises were moved from the middle to the left and right corner of the eyes using Adobe Photoshop. Simulus size was 25 cm×23 cm.

Infants sat on their mother's lap in a dimly lit, sound-attenuated and electrically shielded cabin at a viewing distance of 90 cm away from a 70 Hz 17-inch stimulus monitor. The experiment consisted of one block with 160 trials (containing 80 neutral and 80 fearful face trials). Stimuli were presented using the software ERTS (BeriSoft Corporation, Germany). The two conditions were presented to the infant in a random order with the constraint that the same condition was not presented three times consecutively and that the number of presentations of each set of stimuli was balanced in every 16 trials. The same faces and objects were presented in each of the experiments and in neutral and fearful trials, respectively. Only trials were included in which the infant had seen both the face-object stimulus and the following object. If the infant became fussy or uninterested in the stimuli, the experimenter gave the infant a short break. The session ended when the infant's attention could no longer be attracted to the screen. EEG was recorded continuously and the behavior of the infants was also video-recorded throughout the session.

### Electrophysiological recordings

The same methods and statistical analyses were applied for each of the three experiments. EEG was recorded continuously with Ag-AgCl electrodes from 23 scalp locations of the 10–20 system, referenced to the vertex (Cz) which were attached to a cap. Data were amplified via a Twente Medical Systems 32-channel REFA amplifier. Horizontal and vertical electro-oculograms were recorded bipolarly. Sampling rate was set at 250 Hz. EEG data were re-referenced offline to the linked mastoids. A bandpass filter was set from 0.3–20 Hz.

The EEG recordings were segmented into epochs of waveform that comprised a 200 ms baseline and 1000 ms of one static image featuring an object. For the elimination of electrical artifacts caused by eye and body movements, EEG data were rejected off-line whenever the standard deviation within a 200 ms gliding window exceeded 80 µV at EOG electrodes or 50 µV at any scalp electrode. Data were also visually edited offline for artifacts and matched with the infant's recorded behavior. Only trials were included in which the infant had looked to the screen and displayed no eye movements.
